# Association between length of residence and overweight among adult immigrants in Portugal: A nationwide cross-sectional study

**DOI:** 10.1186/s12889-017-4252-5

**Published:** 2017-04-13

**Authors:** Liliane Peralta da Costa, Sónia Ferreira Dias, Maria do Rosário Oliveira Martins

**Affiliations:** Global Health and Tropical Medicine, GHTM, Instituto de Higiene e Medicina Tropical, IHMT, Universidade Nova de Lisboa, UNL, Rua da Junqueira 100, 1349–008 Lisbon, Portugal

**Keywords:** Immigrants, Portuguese population, Length of residence, Overweight

## Abstract

**Background:**

Despite the importance of immigrant population in Portugal few studies have analyzed the patterns of overweight/obesity in this subpopulation. The aims of this study are: (i) describe and compare the prevalence of overweight between immigrants and natives in Portugal; (ii) analyze the association between length of residence and overweight among adult immigrants in Portugal.

**Methods:**

A cross-sectional study (2005–2006) in a representative sample of the Portuguese population from national territory, including the Autonomous Regions of Azores and Madeira. The final sample comprised 31,685 adult participants (≥19 years old), of whom 4.6% were immigrants. Country of birth was used to determine immigrant condition. Logistic regressions were conducted to investigate the association between overweight (dependent variable) and length of residence (exposure), adjusting for all covariates in the study. A 5% confidence level and 95% CI were considered.

**Results:**

The percentage of immigrants that are overweight [44.9% (95% CI: 42.3; 47.5)] was lower than for natives [52.8% (95% CI: 52.2; 53.4)]. The migrant condition, after adjusted for sociodemographic variables, was not associated with overweight [OR 1.004 (95% CI: 0.998; 1.010)]. Among immigrants, being women [OR 0.585 (95% CI: 0.583; 0.587)], not married [OR 0.784 (95% CI: 0.781; 0.787)] and with a higher education [OR 0.481 (95% CI: 0.478; 0.483)], are probably protective factors of being overweight. Adjusting for other factors, the odds of being overweight for a long-term immigrant (≥15 years) was 1.3 times higher [OR 1.274 (95% CI: 1.250; 1.299)] than for the newcomers (<4 years).

**Conclusions:**

The prevalence of overweight was higher for natives than immigrants. Length of residence (≥15 years) was positively associated with prevalence of overweight, among adult immigrant population. In the future, understanding dietary patterns and acculturation process may be important for health immigrant studies.

## Background

Migration is a global phenomenon and it has been identified in the literature as a social determinant of health [1]. Obesity is particular relevant because migrants from low-income countries tend to adopt health behaviors that may differ from those of the country of origin, such as a westernized, energy-rich diet and more sedentary lifestyles [1, 2]. In Europe, information available on migrants health is scarce which constitute a challenge to monitor and improve migrant health outcomes [1].

At present, immigrants are a valuable population for the Member States of the European Union due on one hand to the potential for population growth engine to counter the aging population and low fertility rates, and on the other hand, the economic potential to work given the shortage of labor [1, 3–5]. Recognition of the migration role in economic development and sustainability emphasizes the subject of health of these populations as one of the priorities for health policies [1, 5].

Worldwide obesity, defined as abnormal or excessive fat accumulation that presents a risk to health, has more than doubled since 1980. The rise of Body Mass Index (BMI), a simple index of weight-for-height that is commonly used to classify overweight and obesity in adults, is a major risk for non-communicable diseases such as cardiovascular diseases, diabetes, musculoskeletal disorders and some cancers [6]. Between 1980 and 2013, the prevalence of overweight and obesity combined rose by 27.5% for adults, worldwide [7]. In 2014 more than 1.9 billion adults, 18 years and older, were overweight. Of these over 600 million were obese [6]. An estimated 35.8 million (2.3%) of global DALYs (*Disability-Adjusted Life Years*), a measure expressed as the sum of years of potential life lost due to premature mortality and years of productive life lost due to disability, are caused by overweight and obesity [8].

World Health Organization (WHO) Regions have different rates of overweight. The prevalence of overweight in both sexes is higher in WHO Regions of the Americas (62%) and lower in WHO Region for South East Asia (14%) [8]. Data from the 53 countries in WHO European Region revealed that, if we consider both sexes, more than 50% of the adult population is overweight [9]. According to countries profiles, the prevalence of overweight was higher in Czech Republic (72%) and Turkey (64%) among adult males and females, respectively. Tajikistan had the lower rates of overweight in both sexes. Portugal, with 59.1% of overweight adults, is ranked in position 17, just below Malta (64.3%) and Spain (62.0%), and above Greece (53.7%), Italy (54.1%) and France (50.7%) [9]. Worldwide, the proportion of adults overweight increased between 1980 and 2013 from 28.8% to 36.9% in men, and from 29.8% to 38.0% in women [7].

Length of residence in the host country, often a measure of acculturation, is an important determinant of immigrants’ health [10, 11]. The effect of the migration process on BMI appears to be negative and the odds of overweight/obesity increase with length of residence [11]. This assumption is limited, however, by empirical inconsistencies and methodological issues [12, 13]. In Europe, studies that investigate the impact of length of residence in weight status are fewer and show mixed results among themselves and when compared to studies with United States of America (USA) immigrants [11].

The literature shows that length of residence in the USA was significantly and positively associated with overweight/obesity, although this relationship seems to vary according to ethnic groups [14–17]. In Canada the results are similar to those found in the USA [10, 18]. For most immigrants in Canada the probability of becoming overweight is lower on arrival than for comparable native-born Canadians, but increases gradually with additional years in the new country. In Norway [19], BMI of African immigrants increases with length of residence, but in France [20] this result is evident only among older men. Other studies, conducted in Spain and Netherland, report no positive association between length of residence and the prevalence of overweight/obesity or that it disappears after adjusting for sociodemographic variables and health status [21, 22]. In Sweden and Switzerland prevalence of overweight/obesity is higher for immigrants than for natives [23, 24].

Despite the importance of immigrant population in Portugal [5, 25], few studies have analyzed the patterns of overweight/obesity in this subpopulation. Using data from the National Health Survey (NHS) this study: (i) describes and compares the prevalence of overweight between immigrants and natives in Portugal; (ii) analyze the association between length of residence and overweight among adult immigrants in Portugal.

## Methods

### Population and sample

Data used in this study come from the fourth edition of the National Health Survey (4th NHS), a representative survey of the Portuguese population, planned and conducted by the Statistics Portugal and the National Institute of Health Dr. Ricardo Jorge (INSA), in collaboration with the Directorate-General of Health [26]. The 4th NHS collected information on health status, health determinants, use of health services, and sociodemographic characteristics of individuals and was the first edition to cover the entire national territory, including the Autonomous Regions of Azores and Madeira. Also for the first time, data refers to all resident population, regardless of their nationality or migrant status. The questionnaire was administered by direct interview to a representative probability sample of the Portuguese population between February 2005 and February 2006. The study population included individuals living in family housing units (thereby excluding people living in collective accommodation). A total of 41,193 individuals living in 15,239 family housing units were interviewed, and the interview completion rate was 76% nationwide. The NHS methodology has been previously described elsewhere [26].

Country of birth was used to determine immigrant condition and we consider only adult population (age > 19 years). We excluded participants with missing information and also those with unreliable BMI (≤12.6 or ≥39.3, *n* = 292). The final sample size comprised 31,685 participants, of whom 95.4% (*n* = 30,238) were Portuguese and 4.6% (*n* = 1447) were immigrants.

### Measures

BMI was calculated based on self-reported height and weight data, and categorized according to WHO as pre-obesity (25.0–29.9 kg/m^2^) and obesity (≥30.0 kg/m^2^) [27]. The outcome variable is overweight, defined as a binary variable that is equal to one if BMI ≥25.0 kg/m^2^ and zero otherwise.

Demographic characteristics included gender, age (20 to 24; 25 to 34; 35 to 44; 45 to 54; 55 to 64 and ≥65 years) and marital status (married, not married). Socioeconomic status was measured by educational level (none or 1^st^cycle, 2nd or 3th cycle, high school, and post-secondary or higher education), and job status (active, unemployed, other). Immigrants were grouped in regions according to their country of origin (Europe, Africa, America or Asia). Smoking status was also included as control variable: currently smoke cigarettes daily or occasionally; no current smoker.

Finally, acculturation and new life styles were measured by the length of residence, in years, and categorized as follows: <1; 1 to 4; 5 to 9; 10 to 14 and ≥15 years. Similar cut-points have been used in previous research [15, 17, 28–33]**.** Due to sample size constrains the first two categories were collapsed resulting in four categories.

### Statistical analysis

Descriptive analysis for both sociodemographic and anthropometric characteristics were conducted for immigrants and natives; for the subsample of immigrants, acculturation characteristics were also provided by gender, age, education, marital status, smoking status and region of origin.

To compare overweight between immigrants and natives we used descriptive statistics (frequencies and graphics) and logistic regression. Using overweight (1 or 0) as dependent variable, we estimate the unadjusted and adjusted odds ratio for the risk factor of being immigrant versus native.

To investigate the association between acculturation and overweight, we used the subsample of immigrants and conducted a logistic regression with overweight as dependent variable and length of residence as exposure, adjusting for possible confounders such as age, education, gender, marital state, job status, smoking status and region of origin [34].

We considered a 5% confidence level and 95% confidence interval (CI). The data analysis was performed using statistical package IBM SPSS®, version 22.0.

## Results

Characteristics of the study population, native and immigrants, are described in Table 1. Immigrants were younger, more educated, and presented a higher employment activity rate when compared with natives. The percentage of immigrants that were overweight [44.9% (95% CI: 42.3; 47.5)] was lower than for natives [52.8% (95% CI: 52.2; 53.4)].

**Table 1:** Sociodemographic characteristics and prevalence of pre-obesity/obesity of native and immigrant population by region of origin

	Portuguese	Immigrants	by region		
	*N* = 30,238	*N* = 1447	Europe	Africa	America/Asia
			*n* = 462	*n* = 644	*n* = 341
Gender (%)					
Men	47.6	48.6	44.5	49.7	50.4
Women	52.4	51.4	55.5	50.3	49.6
Age (%)					
20–24	8.5	10.1	15.9	3.8	19.4
25–34	18.6	38.1	51.7	32.0	37.8
34–44	18.6	23.7	21.2	24.4	24.9
45–54	16.9	15.8	6.1	22.7	9.4
55–64	14.7	6.5	2.9	9.0	4.0
≥ 65	22.7	5.8	2.2	7.9	4.5
Marital status (%)					
Married	65.0	59.3	61.1	57.9	60.7
Not married	35.0	40.7	38.9	42.1	39.3
Education (%)					
None or 1st cycle	48.4	17.3	6.2	22.1	18.1
2nd or 3rd cycle	27.2	37.7	35.8	38.0	39.9
High school	11.5	25.3	31.2	23.7	22.8
Higher education	12.9	19.3	26.8	16.1	19.2
Job status (%)					
Active	58.5	75.6	78.8	73.1	78.5
Unemployed	5.6	7.7	6.0	8.1	8.6
Other^a^	35.7	16.6	15.2	18.8	12.9
Current smoker (%)	21.0	26.2	30.7	27.4	18.1
Median Length of Residence (years)		19	16	28	5
BMI (%)					
Pre-obesity	37.5	33.1	30.2	36.4	27.9
Obesity	15.3	11.8	9.0	13.0	12.0

^a^Pensioners, students, housewives, permanently incapacitated, unpaid internship, or other status

For immigrant population, the median length of residence was 19 years, ranged from less than 1 to 76 years. Most of the immigrants were from Africa [54.2% (95% CI: 51.6; 56.8)], was less than 45 years old [71.9% (95% CI: 69.6; 74.2)], was married [59.3% (95% CI: 56.8; 61.8)], well educated [44.6% with high school or higher (95% CI: 42.0; 47.2)], and worked [75.6% (95% CI: 73.4; 77.8)]. The characteristics of immigrants by length of residence are in Table 2.

**Table 2:** Characteristics and prevalence of pre-obesity/obesity of immigrant population by length of residence in Portugal

	Immigrants			
	<4 y (*n* = 245)	5–9 y (*n* = 197)	10–14 y (*n* = 140)	≥15 y (*n* = 31,103)
Gender (%)				
Men	44.6	48.4	59.3	48.0
Women	55.4	51.6	40.7	52.0
Region of origin (%)				
Europe	26.6	24.1	33.8	22.5
Africa	18.5	49.8	51.8	66.5
America/Asia	54.8	26.2	14.5	11.1
Age (%)				
20–24	19.4	12.1	6.2	7.5
25–34	49.7	44.8	34.0	33.6
34–44	17.8	23.1	46.1	21.9
45–54	8.0	11.9	6.3	20.8
55–64	3.9	6.2	7.3	7.2
≥ 65	1.3	1.9	0.1	9.1
Marital status (%)				
Married	59.8	58.7	45.5	61.6
Not married	40.2	41.3	54.5	38.4
Education (%)				
None or 1st cycle	14.8	21.0	15.7	17.4
2nd or 3rd cycle	45.1	36.1	38.7	36.0
High school	27.2	26.1	26.0	24.5
Higher education	13.0	16.7	19.6	22.0
Job status (%)				
Active	71.1	81.6	83.9	74.1
Unemployed	11.8	5.6	4.5	7.6
Other^a^	17.1	12.8	11.6	18.4
Current smoker (%)	18.8	28.2	26.2	28.0
BMI (%)				
Overweight	37.5	37.9	39.1	48.8
Pre-obesity	26.1	31.4	39.9	34.4
Obesity	11.4	6.5	5.2	14.4

^a^Pensioners, students, housewives, permanently incapacitated, unpaid internship, or other status

Analysis by length of residence in Portugal reveals that newcomers or recent immigrants (<4 years) were younger than long-term immigrants (≥15 years), and had less frequency of current smoking habits [18.8% (95% CI: 13.9; 23.7)] (Table 2). Most immigrants who had been in Portugal for five or more years were from Africa, but the latest immigration wave were mainly from America or Asia [54.8% (95% CI: 48.6; 61.0) for <4 years].

This study reveals that the overweight prevalence increased with length of residence in Portugal [37.5% (95% CI: 31.4; 43.6) among recent immigrants and 48.8% (95% CI: 48.2; 49.4) among those living in the country for ≥15 years]. The prevalence of pre-obesity and obesity was higher among immigrants living in Portugal for 10–14 years [39.9% (95% CI: 31.8; 48.0)] and for ≥15 years [14.4% (95% CI: 14.0; 14.8)], respectively.

**Fig. 1:**
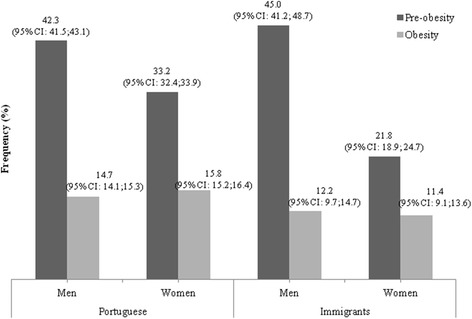
Prevalence (%) of pre-obesity and obesity by gender, among Portuguese and immigrant populations

Figure 1 shows overweight prevalence stratified by gender. Men had higher prevalence of overweight (either pre-obesity or obesity) than women, in both native and immigrant population. The exception was for the prevalence of obesity in the Portuguese population, which was higher in women [15.8% (95% CI: 15.2; 16.4) of Portuguese women were obese].

Table 3 shows results from two logistic regression models. In model 1 we used the whole data set to estimate the association between being overweight and the migrant condition, adjusting for all other possible factors. In model 2, we restricted our analysis to immigrants and we investigated the association between being overweight and length of residence, adjusting for other factors.

**Table 3:** Association between being overweight and migrant condition (Model 1) and length of residence (Model 2)

	Model 1		Model 2	
	Crude	Adjusted	Crude	Adjusted
	OR (95% CI)	OR (95% CI)	OR (95% CI)	OR (95% CI)
Migrant condition				
Native	1 (reference)	1 (reference)		
Immigrant	0.729 (0.724;0.733)	1.004 (0.998; 1.010) †		
Length of residence				
< 4 y.			Reference	Reference
5–9 y.			1.016 (0.996; 1.037)	0.785 (0.767; 0.803)
10–14 y.			1.373 (1.342; 1.404)	0.995 (0.970; 1.022) †
≥ 15 y.			1.599 (1.575;1.624)	1.274 (1.250; 1.299)
Gender				
Men		1 (reference)		Reference
Women		0.585 (0.583; 0.587)		0.321 (0.317; 0.325)
Age				
20–24 y.o.		1 (reference)		Reference
25–34 y.o.		2.055 (2.041; 2.069)		4.303 (4.178; 4.331)
35-44y.o.		2.662 (2.643; 2.681)		4.277 (4.145; 4.413)
45–54 y.o.		4.099 (4.069; 4.130)		7.322 (7.082; 7.571)
55–64 y.o.		4.449 (4.415; 4.484)		9.697 (9.334; 10.074)
≥ 65 y.o		3.116 (3.092; 3.140)		5.856 (5.623; 6.100)
Marital status				
Married		1 (reference)		Reference
Not married		0.784 (0.781; 0.787)		0.756 (0.746; 0.766)
Education				
None or 1st cycle		1 (reference)		Reference
2nd or 3rd		0.725 (0.722; 0.727)		0.502 (0.493; 0.512)
High school		0.603 (0.600; 0.607)		0.456 (0.456; 0.465)
Higher education		0.481 (0.478; 0.483)		0.399 (0.391; 0.408)
Job status				
Active		1 (reference)		Reference
Unemployed		1.009 (1.002; 1.015)		0.495 (0.483; 0.507)
Other^*^		1.013 (1.009; 1.017)		0.662 (0.647; 0.676)
Current smoker		1.699 (1.693; 1.706)		1.271 (1.253; 1.289)
Region of origin				
Europe				Reference
Africa				0.999 (0.983; 1.015) †
America/Asia				0.918 (0.900; 0.936)

^*^Pensioners, students, housewives, permanently incapacitated, unpaid internship, or other status; † *p* > 0.05

Being immigrant, after adjusted for sociodemographic variables, was not associated with overweight [OR 1.004 (95% CI: 0.998; 1.010)] (model 1). Among immigrants, being women [OR 0.585 (95% CI: 0.583; 0.587)], not married [OR 0.784 (95% CI: 0.781; 0.787)] and with a higher education [OR 0.481 (95% CI: 0.478; 0.483)], are probably protective factors of being overweight (model 2). However, the length of residence appears as a risk factor: adjusting for other factors, the odds of being overweight for a long-term immigrant (living for 15 years or more in Portugal) was 1.3 times higher [OR 1.274 (95% CI: 1.250; 1.299)] than for the newcomers (<4 years). The African origin and 10–14 years of length of residence were not statistically associated with the probability of being overweight.

## Discussion

Among adults in Portugal, the prevalence of overweight was higher for natives than immigrants, which is in line with the “Healthy Immigrant Effect” (HIE), a phenomenon in which immigrants are on average healthier than the native born [2]. Some explanations for the HIE include health screening by immigration officers, relatively healthier behaviors of new immigrants prior to migration, and immigrant self-selection whereby the healthiest and wealthiest individuals are the people most likely to migrate [2]. This finding is in line with other research conducted in Portugal, showing an immigrants’ health advantage in terms of chronic conditions for some groups of immigrants [35] and a lower prevalence of pre-obesity and obesity among Brazilian and African immigrants, compared with the Portuguese population [36]. Immigrants from this study were an active, educated, and younger population, which could explain the lower levels of overweight. It is reasonable to assume that they are in a better position to take advantage of educational initiatives and health information than their less active, less educated, and older counterparts. Data and participants were selected from accommodations units, which constitute a limitation of the NHS that may explain these results. Therefore, it is possible that the sample did not consider vulnerable groups, namely refuges or irregular immigrants, with different socioeconomic characteristics and health outcomes. The report on health of migrants in Portugal reinforces this effect of selection: the description of health determinants revealed higher food insecurity for Portuguese population; among immigrants, the higher frequency of food restriction due to economic difficulties was reported by recent immigrants (≤5 years) [37].

In Portugal, for long-term immigrants (≥15 years), the odds of being overweight increased. This loss of health advantage with length of residence in the host country is attributed in the literature to lifestyle changes, including patterns of physical activity and dietary habits, that describe the phenomena of nutrition transition and dietary acculturation [10, 38]. The concept of nutrition transition focuses on large shifts in diet and physical activity patterns, due to globalization and urbanization, and influences nutritional outcomes, such as changes in average stature, body composition, and morbidity [39]. Dietary acculturation refers to the process that occurs when members of a migrating group adopt the eating patterns/food choices of their new environment [40]. Looking beyond the overall tendencies, the relationship between overweight and acculturation is complex. Acculturation is a multidimensional and dynamic process, but not necessarily deleterious. Immigrants may retain traditional foods, exclude others, and find new ways to use traditional foods or adopt the diet pattern of the host country. Despite this, in general dietary acculturation has been found to have detrimental effects on diets of immigrants and racial/ethnics minorities [41]. Length of residence may not fully capture the acculturation process, but it is considered a reasonable surrogate measure and has been used in many studies [10, 11, 42].

Nutrition transition is accelerated by migration [43] but promotion of the consumption of traditional foods and retention of cultural eating patterns are known to partially prevent this transition [44]. Food culture arises out of the place of a people’s origin and is shaped by resources (climate, land, soil, water, and fuel), belief and information (religion, education and literacy and communication), ethnicity, technology, colonization, health status, and health care [44]. In the new food environment, socioeconomic and demographic characteristics (e.g. age, gender, education, employment status, language, religion, household composition, income, food availability and accessibility, and place of residence), ethno-cultural norms, political-economic process, and exposure to the host culture (e.g. access to media, peers, and access to traditional supermarkets) may influence dietary choices [41, 45]. Because migrant populations are heterogeneous and move through different phases of the health transition during their life course, researchers studying migrant health should consider risks and exposures not only in the host country, but also during the migration process and in the country of origin [46]. Nutrition transition level in the country of origin is important because of the accelerated pace of changes in diet and physical activity patterns observed in low and middle-income countries [39, 47, 48]. Other important factors include genetic background, health behavior like nutrition, physical activity, and alcohol and tobacco consumption [46]. In line with this, knowledge of traditional food habits and study of health determinants, before and after migration, may be necessary to understand how dietary acculturation could potentially reverse current trends in overweight and to plan intervention policies to improve the health of migrants and natives.

Most studies that confirm a positive association between length of residence and overweight report a threshold effect of duration on weight gain after 10–15 years of residence [14]. This study found the strongest positive association after 15 years in the host country. These results may provide some evidence that immigrants in Portugal maintain their food habits (probably less obesogenic) for a few years, until they adopt (especially males) a more obesogenic behavior (diet and physical activity patterns). To confirm this theory, further research will have to address the characteristics of dietary pattern, upon arrival in the host country and over time. Dietary pattern analysis has emerged as an alternative and complementary approach to examining the relationship between diet and the risk of chronic diseases [49]. Instead of looking at individual nutrients or foods, pattern analysis examines the effects of overall diet. Conceptually, dietary patterns represent a broader picture of food and nutrient consumption, and may thus be more predictive of disease risk than individual foods or nutrients.

The overweight prevalence (60.1%) found by Goulão et al. [36], among Brazilian and African immigrants settled in Portugal for ≥15 years, was higher than the prevalence found in this study (48.8%) for the same time of residence. One possible explanation is that Goulão et al. included irregular immigrants living in enclaves, which may have different socioeconomics characteristics and thus, poorer dietary patterns and health outcomes. In the present study, among immigrant population the prevalence of overweight was higher for Africans (49.4%). One possible explanation is that Africans were living in Portugal for a long time (28 years as median of length of residence). More surprisingly was the high prevalence of overweight within American/Asian (39.9%), notwithstanding that half of this population were in Portugal for only 5 years. According to the Portuguese Immigration Services, the Brazilian community remains the main foreign resident community in Portugal [50]. Therefore, investigating the nutrition transition of Brazil may be important to understand how time of exposure to the host environment and dietary acculturation process may influence overweight.

According to Popkin (2010), belonging to the lower socioeconomic group has conferred strong protection against obesity in low-income countries, but it is a systematic risk factor in upper- to middle-income countries. In Brazil, there is solid evidence that the burden of obesity is shifting toward the poor and, among women is increasing among lower income groups and falling among those in higher income groups [51]. A decrease in the incidence of obesity in Brazilian women was observed from 1989 to 2003, and was seen as related to an increase in income or educational achievement, particularly in the urban setting [52]. Therefore, it is possible that the wave of immigrants from Brazil that arrived in Portugal, because of the intermediate stage of nutrition transition of country of origin, were already overweight, and that the prevalence of this disease is not explained by acculturation. Frequency of American/Asian immigrants that were in Portugal for <4 years (54.8%) may support this statement. These results highlight the importance of other health and nutritional determinants in longitudinal immigrant studies.

In the present study, compared to men immigrant women are less likely to be overweight, which is not consistent with other studies, in which women are found to be more susceptible to being overweight than men, because of their vulnerability to the acculturation process [14, 53]. The explanation for these differences may lie in social factors like gender roles and relations. Among immigrants in Portugal, married males, less educated and 45–54 years old, may constitute a risk group for overweight.

In developed countries men tend to have a higher prevalence of overweight and obesity than women whereas the opposite is more frequently in developing countries [7]. In this study, aggregating countries into only three regions may dissimulate important differences between and within countries. Knowledge about country of origin could give some insight on the subject.

The present study has some limitations. Data from NHS were collected in 2005/2006 and the situation may change at this time. Nevertheless, it was the first to include the entire resident population, regardless origin country, so these results may serve as comparison with future studies. Another limitation is that data were aggregate in just three regions, one of which, America/Asia, has the highest and the lowest overweight prevalence among all WHO regions in 2008 [8]. Factors related to diet or physical pattern were not adequately considered and no questions were included in the NHS to address dietary acculturation. The cross-sectional nature prevents us from making any inferences regarding causality and height and weight were self-reported, with all biases associated with self-reported data.

The main strength of this study is to highlight the importance of nutritional and dietary assessment of immigrants upon arrival in the host country and over time. Future studies on immigrants’ health should consider dietary patterns analysis, information about country of origin nutrition transition level, and measures that could identify degrees of acculturation across several domains, especially dietary acculturation. Further research of the association between migration and weight status should involve longitudinal studies of immigrants from different ethnic backgrounds, as well as instruments that more accurately measure the various steps in the process of dietary acculturation.

## Conclusions

Among adults in Portugal, the prevalence of overweight was higher for natives than immigrants. Length of residence (≥15 years) was positively associated with prevalence of overweight among adult immigrant population. In the future, understanding the role of dietary patterns on the acculturation process may be important to prevent health deterioration with longer residency of immigrants in Portugal.

## Abbreviations

BMI: Body Mass Index
CI: Confidence Interval.
DALYs: Disability-Adjusted Life Years
HIE: Healthy Immigrant Effect.
INSA: Statistics Portugal and the National Institute of Health Dr. Ricardo Jorge
NHS: National Health Survey.
USA: United States of America.
WHO: World Health Organization
